# Physiological and Transcriptomic Evaluation of Drought Tolerance in Alfalfa (*Medicago sativa* L.) and Identification of Resilient Germplasm

**DOI:** 10.3390/plants15111737

**Published:** 2026-06-03

**Authors:** Lixin Sun, Juan Zhou, Xiaoyan Zhao, Hongxia Ding, Rui Ma, Minshan Sun, Feng Wei

**Affiliations:** 1 Yinchuan Forestry, Grassland, and Landscape Management Bureau, Yinchuan 750021, China; 13619512322@163.com; 2 College of Ecology, Environment and Chemical Engineering, Hetao College, Bayannur 015000, China; zhoujunanlee@163.com; 3 College of Enology and Horticulture, Ningxia University, Yinchuan 750021, China; xuguo120826@163.com (X.Z.); outsource2024@163.com (H.D.); novebio@163.com (R.M.); 4 College of Plant Protection, Henan Agricultural University, Zhengzhou 450002, China

**Keywords:** alfalfa, drought stress, leaf and root, physiological characteristics, comprehensive evaluation

## Abstract

Drought stress is a major constraint on alfalfa (*Medicago sativa* L.) production. Screening for drought tolerance at the seedling stage can accelerate the identification of resilient germplasm. In this study, six alfalfa cultivars were selected and subjected to drought stress at the seedling stage. Morphological traits (stem diameter, plant height, biomass, and root–shoot ratio) and oxidative/antioxidant indicators (malondialdehyde (MDA), superoxide (O_2_•^−^), superoxide dismutase (SOD), peroxidase (POD), catalase (CAT), glutathione reductase (GR), and ascorbate peroxidase (APX) activity) were quantified in leaves and roots. Drought stress significantly reduced plant height (by up to 42.4% in ZL2) and biomass (by up to 30% in some cultivars), but increased the root–shoot ratio (by 50–166%). MDA and O_2_•^−^ levels increased by 10–174% in leaves and 8–65% in roots across cultivars. Antioxidant enzyme activities rose markedly: SOD by 23–125% in leaves and 2–100% in roots; POD by 47–240% (leaves) and 38–166% (roots); CAT by 9–129% (leaves) and 30–227% (roots); GR by 35–107% (leaves) and 23–172% (roots); APX by 8–175% (leaves) and 3–89% (roots), indicating a coordinated leaf–root antioxidant defense. Transcriptome analysis of the tolerant cultivar ZM3 revealed 853 differentially expressed genes, which were enriched in pathways such as the non-homologous end-joining DNA repair pathway. Multivariate assessment of seedling-stage performance identified ZM3 and ZL2 as the most drought-tolerant cultivars. Collectively, these findings provide germplasm leads and empirical evidence for coordinated leaf–root antioxidant strategies in alfalfa, informing the selection and improvement of drought-tolerant cultivars.

## 1. Introduction

Drought stress is a major environmental threat affecting plants in arid and semi-arid regions [1,2]. It significantly reduces crop yield and can even cause plant death under severe conditions. As climate change progresses, drought stress has intensified across multiple regions, and the expansion of drought-affected areas has exerted a pronounced negative impact on global crop productivity [1]. However, different plants respond differently to drought stress, and certain species can maintain good growth even under drought conditions, demonstrating strong resistance. Therefore, investigating the physiological and ecological changes in plants under limited water availability is essential for developing drought-resistant crops. Numerous scholars have conducted in-depth studies on the growth patterns, physiological responses, and biochemical mechanisms of forage plants under drought stress. Research indicates that these plants adapt to drought by modulating metabolic rates, endogenous hormone levels, and carbon allocation patterns, as well as by accumulating osmotic regulatory substances and enhancing antioxidant enzyme activity [2].

Abiotic stresses, such as drought, significantly impact plant physiological activities. For example, drought reduces leaf area, water potential, and photosynthetic rate, and also slows material transport within the plant [3]. Additionally, it induces a sharp increase in reactive oxygen species (ROS) content, leading to oxidative damage, which can lead to plant death under severe conditions [4]. Under drought stress, growth indicators such as biomass, plant height, and root-to-shoot ratio (RSR) often decline, while the root structure undergoes adaptive changes. Increased lateral root number and root thickening alter carbon allocation patterns, limiting aboveground biomass accumulation and reducing water transpiration losses [5]. Meanwhile, lateral root growth expands the contact area of the root system with the soil, enhancing water absorption and utilization [6]. Leaves, as key organs for photosynthesis and transpiration, exhibit external morphological characteristics that directly reflect the extent of drought response and are closely associated with water deficit. During the early stages of drought stress, stomata on plant leaves close to limit water loss owing to transpiration, while the leaves show reduced thickness and decreased water content [7]. Together, these morphological and physiological changes play a crucial role in enhancing water retention capacity, reducing transpiration losses, and maintaining photosynthetic efficiency.

Accumulation of osmotic regulatory substances and enhancement of antioxidant capacity are two key physiological mechanisms by which plants respond to drought stress. Plants accumulate Ca^2+^, proline, sorbitol, betaine, soluble sugars, and soluble proteins to reduce cellular osmotic potential, maintain osmotic balance, and thereby mitigate damage caused by osmotic stress [8]. However, drought stress induces a sharp increase in reactive ROS levels, leading to oxidative damage, which can be severe enough to cause plant death [9]. To effectively reduce ROS production, plants have evolved complex and precise antioxidant systems. Among these, antioxidant enzymes such as catalase (CAT), peroxidase (POD), superoxide dismutase (SOD), and ascorbate peroxidase (APX) play a vital role in ROS scavenging [10]. Additionally, drought stress signals are systematically transmitted within plants through the synthesis and regulation of endogenous hormones, enabling plants to obtain nutrients and enhance their drought tolerance.

In summary, drought tolerance is a complex, multifaceted trait involving multiple interrelated physiological and biochemical processes that are integrated across all stages of plant growth and development [11]. To gain a deeper understanding of drought tolerance mechanisms and capacity in plants, a comprehensive analysis of physiological and growth factors through experimentation is necessary.

Alfalfa (*Medicago sativa* L.) is a high-yielding, nutrient-rich, and stress-tolerant perennial legume, often referred to as the “king of forage crops” because of its high palatability, digestibility, and economic value [12,13]. Its well-developed deep root system and symbiotic relationship with rhizobia form an efficient nitrogen-fixing system that improves soil structure and contributes to soil and water conservation in semi-arid regions [14]. In China, alfalfa is primarily cultivated in arid and semi-arid regions, where drought is a key factor limiting its growth, yield, and survival [15]. With the intensification of climate change, the adverse impact of drought stress is becoming increasingly evident. Therefore, breeding alfalfa germplasms with high stress tolerance is of critical importance for the sustainable development of the livestock industry in northern China.

To address this issue, we analyzed six representative alfalfa cultivars under simulated drought conditions in a greenhouse. Changes in growth traits (plant height, stem diameter, and biomass) and physiological indicators in leaves and roots were comprehensively evaluated to screen out drought-tolerant cultivars. The aim was to clarify cultivar-specific growth and physiological response patterns at the leaf and root levels under drought stress. Our findings provide a theoretical basis for understanding the synergistic drought resistance strategies of alfalfa leaves and roots for breeding high-yielding, drought-tolerant cultivars.

## 2. Materials and Methods

### 2.1. Experimental Design

Six alfalfa cultivars were selected for this study: Zhongmu No. 3 (ZM3) and Zhonglan No. 2 (ZL2), bred in China; Algonquin (AL), a North American cultivar; DF310, a commercial variety; Qiji (QJ), a local landrace from Gansu; and Gannong No. 4 (GN4), also from Gansu. Seeds were purchased from Ningxia Grass Industry Technology Co., Ltd. (Wuzhong, China), and selected based on their fullness and uniform size. The seeds were washed thoroughly with distilled water and surface-sterilized with 75% (*v*/*v*) alcohol for 5 min. They were then rinsed 3–5 times with sterile distilled water to completely remove residual alcohol. The sterilized seeds were placed on a culture dish lined with moist sterile filter paper and incubated in the dark at 4 °C for 2–3 d to promote germination. Upon germination, 6–8 uniform and vigorous seedlings were selected and transplanted into pots (14 × 16 × 22 cm) containing a substrate mixture of nutrient soil, perlite, and vermiculite (4:1:0.5). The seedlings were grown for 4 w in a greenhouse with 70–75% relative humidity and an average temperature of 25 °C under a 14 h light/10 h dark cycle. After stabilization, the seedlings were subjected to natural drought stress. For the drought stress (DS) group, watering was completely withheld, and the natural drought was sustained for 7 days until the soil moisture reached 30–35% of field capacity, which is widely recognized as a moderate-to-severe drought stress level for pot-grown alfalfa seedlings, as monitored by daily weighing. The control (CK) group was irrigated daily to maintain the soil moisture at 75–80% field capacity. The experiment was arranged in a completely randomized design with two factors (cultivar and water treatment). Each treatment had 3 biological replicates (i.e., 3 pots), totaling 36 pots. On the 7th day after the onset of drought treatment, plant samples were collected to measure growth indices, MDA and O_2_•^−^ content, and the activities of key antioxidant enzymes.

### 2.2. Determination of Various Indicators

#### 2.2.1. Calculation of Plant Height, Stem Thickness, Plant Fresh Weight, and Plant Dry Weight

After 7 d of treatment, leaf height was measured with a ruler in the normal growth state (plant height), while stem thickness was measured using a Vernier caliper. Plant materials were collected, and roots were gently rinsed with water to remove soil. The cleaned roots were separated from the aboveground parts, and both were dried using filter paper. The dried samples were placed in an oven at 105 °C for 15 min to kill any remaining microorganisms, followed by drying at 85 °C until a constant weight was reached. Both the above- and below-ground biomass per plant were weighed, and the RSR and total biomass were calculated. RSR = below-ground biomass/above-ground biomass; Biomass = above-ground biomass + below-ground biomass.

#### 2.2.2. Enzyme Extraction and Assay

Following 1 w of drought stress treatment, the leaves and roots of alfalfa seedlings were collected for enzymatic analysis. Approximately 0.2 g of leaf and root tissue was washed and placed in a pre-cooled mortar. Then, 1.6 mL of pre-cooled 50 mmol/L phosphate buffer (pH 7.8) was added, and the tissue was ground into a homogenate on an ice bath. The homogenate was centrifuged at 4 °C and 12,000 rpm for 20 min. SOD activity was determined using the nitro blue tetrazolium method [16], POD activity using the guaiacol colorimetric method [17], and CAT activity using the ultraviolet absorption method [18]. GR activity was determined according to the method of Schaedle et al. [19], and APX activity was measured following the method proposed by Nakano [20]. Specifically, SOD, GR, and APX kits were purchased from Nanjing Jiancheng Bioengineering Institute (Nanjing, China); POD and CAT kits were purchased from Solarbio Science & Technology Co., Ltd. (Beijing, China).

#### 2.2.3. Determination of MDA and O_2_•^−^

Approximately 0.5 g of alfalfa leaf and root tissue was washed and homogenized in 1.6 mL of 10% TCA. The homogenate was centrifuged at 12,000 rpm for 10 min, and the supernatant was collected for analysis. MDA content was measured using the thiobarbituric acid method [21], and O_2_•^−^ content was determined using the hydroxylamine oxidation method [22]. MDA and O_2_^−^ assay kits were also purchased from Nanjing Jiancheng Bioengineering Institute (Nanjing, China) and Solarbio Science & Technology Co., Ltd. (Beijing, China), respectively.

#### 2.2.4. RNA Extraction, Sequencing, and Transcriptome Analysis

The drought-tolerant cultivar ZM3 was used for RNA sequencing. Leaves were collected from the CK and DS groups on day 7 of treatment, with three biological replicates per treatment (each replicate consisting of pooled samples from three pots). Samples were immediately frozen in liquid nitrogen and stored at −80 °C until use.

Total RNA was extracted using the EASYspin Plant RNA Kit (Aidlab Biotech, Beijing, China). Subsequently, the cDNA library for transcriptomic sequencing was constructed and sequenced by APTBIO (Shanghai, China). Paired-end libraries were prepared using an ABclonal mRNA-seq Lib Prep Kit (ABclonal, Wuhan, China) following the manufacturer’s instructions. Adaptor-ligated cDNA was used for PCR amplification, and the PCR products were purified using the AMPure XP system (Beckman Coulter, Inc., Brea, CA, USA). Library quality was assessed using an Agilent Bioanalyzer 4150 system (Agilent Technologies, Inc., Waldbronn, Germany). Sequencing was conducted on the DNBSEQ-RST7-T7 high-throughput sequencing platform (MGI Tech Co., Ltd., Wuhan, China), generating 150 bp raw FASTQ data. Quality filtering of the raw reads was performed using Fastp (v0.18.0) [23], and high-quality reads were mapped to the alfalfa reference genome (version 3, https://doi.org/10.6084/m9.figshare.12327602.v3) [24] using HISAT2 (v2.2.1) [25]. Differential expression analysis was performed using the R package DESeq2 (v1.40.2) [26]. Genes with |Log2FoldChange| ≥ 1 and Padj < 0.05 were identified as DEGs [27]. BLAST2GO (v5.2.5) and Kobas (v3.0) were used for GO terms and KEGG pathway analysis, respectively, while the R package cluster Profiler (v4.20.0) was used for enrichment analysis [28]. The R software (v4.3.1) and TB tools (v2.476) were used for data visualization [24].

#### 2.2.5. Quantitative Real-Time Polymerase Chain Reaction Validation

Total RNA extraction was conducted according to our previous study, and reverse transcription was carried out using Hifair^®^ III 1st Strand cDNA Synthesis SuperMix for qPCR (gDNA Digester Plus; Yesean Biotech, Shanghai, China). The qRT-PCR was performed using the Gene Applied Biosystems^®^ 7500 Fast and TransStart Top Green qPCR SuperMix (TransGen Biotech, Beijing, China). Relative gene expression levels were calculated using the 2^−△△Ct^ method. Each sample was analyzed in three biological replicates, with three technical replicates per experiment.

### 2.3. Data Processing

All data were expressed as mean ± standard deviation (SD) of three biological replicates. Statistical analyses were performed using SPSS 26.0 (IBM Corp., Armonk, NY, USA), and graphs were generated using Origin 2024 (Origin Lab Corp., Northampton, MA, USA). Comparisons between CK and DS within each cultivar were conducted using Student’s *t*-test. Differences among cultivars under the same treatment were analyzed using one-way analysis of variance (ANOVA), followed by Tukey’s honestly significant difference test for multiple comparisons. In all cases, *p* < 0.05 was considered statistically significant. Drought tolerance coefficients were calculated as treatment value/control value for each indicator.

## 3. Results

### 3.1. Effects of Drought Stress on Growth Traits of Alfalfa

**Figure 1:**
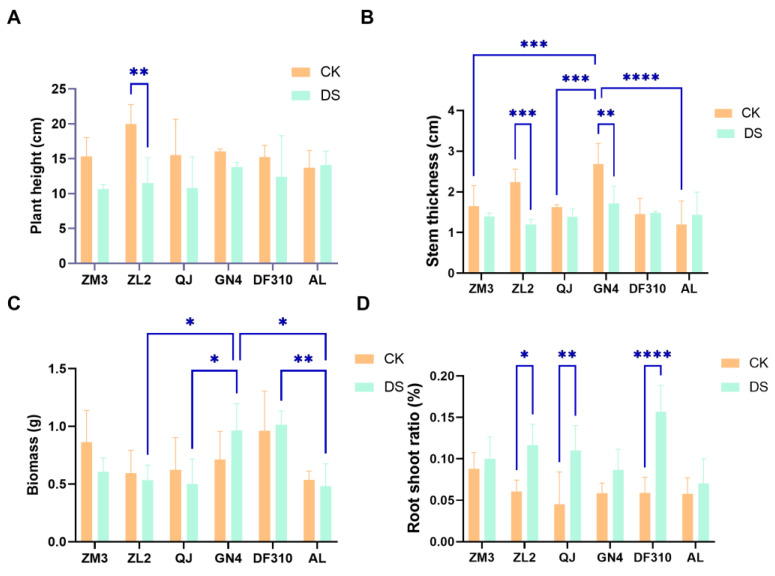
Effects of drought stress on growth traits of six different alfalfa cultivars. (**A**) Plant height. (**B**) Stem thickness. (**C**) Biomass. (**D**) Root shoot ratio. Values are means ± SD (n = 3 biological replicates). Asterisks indicate statistically significant differences as determined by ANOVA analysis. ns, no significance; *, *p* < 0.05; **, *p* < 0.01; ***, *p* < 0.001, ****, *p* < 0.0001.

Drought stress induced significant differences in growth traits among alfalfa cultivars. Overall, drought treatment reduced plant height and biomass, while increasing RSR (Figure 1). Specifically, plant height and biomass in the CK group were higher than those in the DS group, whereas RSR exhibited the opposite trend. Notably, drought stress reduced plant height in all cultivars except AL. The reductions were most pronounced in ZL2 (from 19.96 cm to 11.50 cm, a decrease of 42.4%) and ZM3 (from 15.33 cm to 10.64 cm, a decrease of 30.6%). In contrast, AL showed a slight increase from 13.70 cm to 14.06 cm (Figure 1A). Regarding stem diameter, except for AL and DF310, the other four cultivars (ZM3, ZL2, QJ, and GN4) showed a decreasing trend under DS, with reductions of 15.75% (ZM3), 46.42% (ZL2), 14.19% (QJ), and 36.19% (GN4). Changes in biomass exhibited notable cultivar specificity (Figure 1B). Under DS, biomass varied considerably among cultivars. For example, ZM3 decreased from 0.86 g to 0.60 g, while GN4 increased from 0.71 g to 0.96 g. DF310 showed only a slight increase (from 0.96 g to 1.01 g), representing the smallest change among all cultivars (Figure 1C). The RSR generally increased in all cultivars under drought stress, with DF310 showing the largest increase from 5.89% in CK to 15.67% in DS, significantly higher than other cultivars (Figure 1D). This suggests that DF310 prioritizes resource allocation to the root system under drought conditions, which is a key adaptive strategy to drought stress. Unlike other cultivars, ZM3 showed no significant responses to drought stress, indicating its potentially stronger drought tolerance.

### 3.2. Effects of Drought Stress on MDA and Superoxide Anion (O_2_•^−^) Content in Alfalfa Leaves and Roots

**Figure 2:**
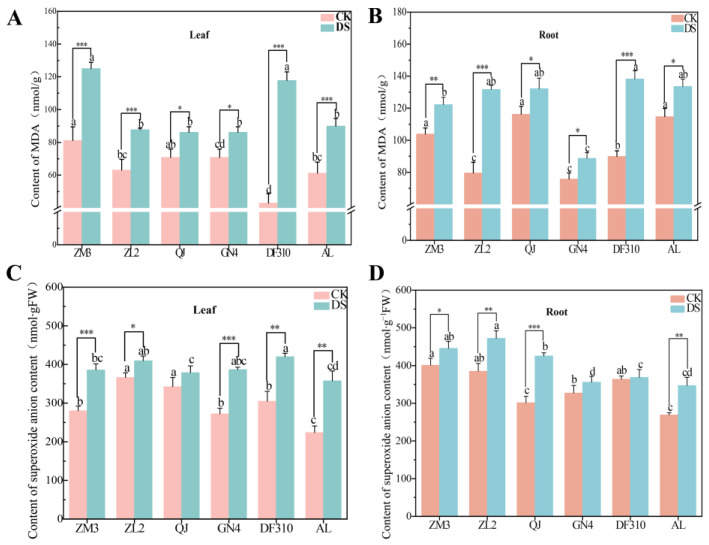
MDA and O_2_•^−^ in leaves and roots of different alfalfa cultivars under drought stress. (**A**) MDA content in leaf. (**B**) MDA content in root. (**C**) O_2_•^−^ content in leaf. (**D**) O_2_•^−^ content in root. Values are means ± SD (n = 3 biological replicates). Asterisks indicate statistically significant differences as determined by *t*-test between CK and DS: ns, no significance; *, *p* < 0.05; **, *p* < 0.01; ***, *p* < 0.001. Roman letters indicate significant differences among different varieties with the same treatments using Tukey’s test for multiple comparisons (*p* < 0.05).

MDA and O_2_•^−^ content are important indicators reflecting membrane damage and lipid peroxidation in plant cells. In DS, MDA content in the leaves and roots of the six alfalfa cultivars significantly increased compared to that in CK (*p* < 0.05 or *p* < 0.001) (Figure 2). In leaves, MDA content increased significantly under drought stress across all cultivars, with increases ranging from 10.35% (ZL2) to 174% (DF310). The largest increases were observed in ZM3 (54.02%), DF310 (174%), and GN4 (76.17%) (*p* ≤ 0.001; Figure 2A). In roots, MDA increases ranged from 16.49% to 65.49% among cultivars, with the highest increase in ZL2 and the lowest in GN4 (Figure 2B). O_2_^−^ levels in leaves increased by 11.77% to 105.62%, with the highest increase in ZM3 and the lowest in QJ (Figure 2C). In roots, O_2_^−^ increases ranged from 8.79% to 41.11%, with the highest in QJ and the lowest in DF3 (Figure 2D). Comparative analysis of the MDA and O_2_^−^ content revealed that in DS, the MDA content in the leaves was generally lower than that in the roots (except in ZM3 and GN4). This suggests that drought stress affects the root system first, causing greater damage in roots than in leaves.

### 3.3. Effects of Drought Stress on Antioxidant Enzyme Activity in Leaves and Roots of Different Alfalfa Cultivars

**Figure 3:**
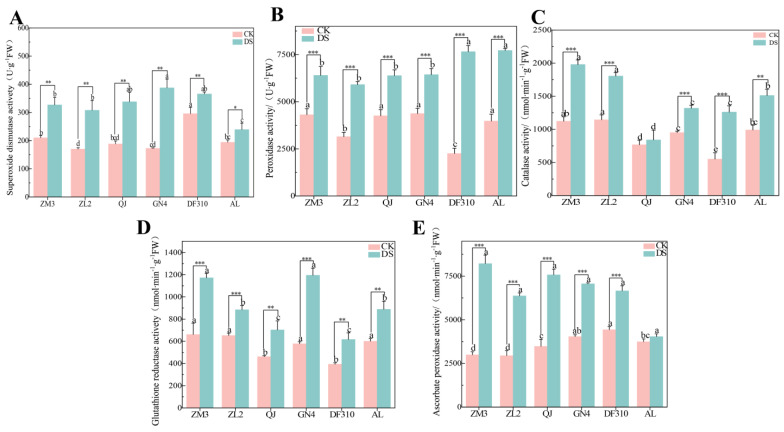
Antioxidant enzyme activity in the leaves of alfalfa seedlings from different cultivars under drought stress. (**A**) Superoxide dismutase. (**B**) Peroxidase. (**C**) Catalase. (**D**) Glutathione reductase. (**E**) Ascorbate peroxidase. Values are means ± SD (n = 3 biological replicates). Asterisks indicate statistically significant differences as determined by *t*-test between CK and DS: ns, no significance; *, *p* < 0.05; **, *p* < 0.01; ***, *p* < 0.001. Roman letters indicate significant differences among different varieties with the same treatments using Tukey’s test for multiple comparisons (*p* < 0.05).

Drought stress leads to excessive accumulation of ROS in plants, causing lipid peroxidation in cell membranes. Typically, plants counteract this by increasing antioxidant enzyme activity to scavenge excess ROS and mitigate oxidative damage [29]. In this study, although the extent of changes varied, the activities of SOD, POD, CAT, GR, and APX in the leaves all showed a significant upward trend in DS compared with those in CK (*p* < 0.05 or *p* < 0.001) (Figure 3). In leaves, drought stress significantly increased the activities of all measured antioxidant enzymes across cultivars, although the magnitude varied. SOD activity increased by 23–125%, with the highest increase in GN4 (124.58%) and the lowest in DF310 and AL (≈23%). ZM3 showed a moderate increase of 55.52% (Figure 3A). POD activity increased by 47–240%, with DF310 showing the largest increase (239.64%) and ZM3 an increase of 48.61% (Figure 3B). CAT activity increased by 9–129%, with DF310 showing the highest (128.88%) and ZM3 a 76.29% increase (Figure 3C). GR activity increased by 35–107%, with GN4 showing the highest (107.12%) and ZM3 a 77.29% increase (Figure 3D). APX activity increased by 8–175%, with ZM3 showing the highest increase (174.90%) and AL the lowest (7.96%) (Figure 3E). This indicates that each alfalfa cultivar relies on its own specific enzymes to enhance drought tolerance, with CAT, GR, and APX being key enzymes for ZM3.

**Figure 4:**
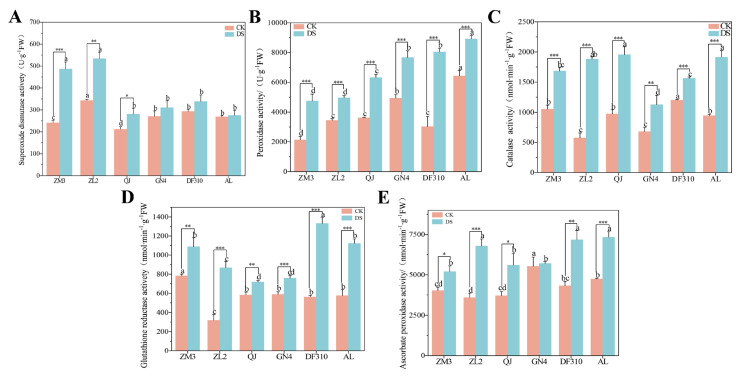
Antioxidant enzyme activity in the roots of alfalfa seedlings from different cultivars under drought stress. (**A**) Superoxide dismutase. (**B**) Peroxidase. (**C**) Catalase. (**D**) Glutathione reductase. (**E**) Ascorbate peroxidase. Values are means ± SD (n = 3 biological replicates). Asterisks indicate statistically significant differences as determined by *t*-test between CK and DS: ns, no significance; *, *p* < 0.05; **, *p* < 0.01; ***, *p* < 0.001. Roman letters indicate significant differences among different varieties with the same treatments using Tukey’s test for multiple comparisons (*p* < 0.05).

In roots, drought stress also significantly enhanced antioxidant enzyme activities across all cultivars. SOD activity increased by 2–100%, with ZM3 showing the largest increase (100.19%) and AL the smallest (2.36%) (Figure 4A). POD activity increased by 38–166%, with DF310 showing the highest (165.64%) and AL a 38.79% increase (Figure 4B). CAT activity increased by 30–227%, with ZL2 showing the largest increase (227.13%) and ZM3 an increase of 60.17% (Figure 4C). GR activity increased by 23–172%, with ZL2 showing the highest (171.51%) and QJ the lowest (23.31%) (Figure 4D). APX activity increased by 3–89%, with ZL2 showing the largest increase (89.36%) and GN4 the smallest (3.23%) (Figure 4E).

### 3.4. Comprehensive Evaluation of Drought Tolerance in Different Alfalfa Cultivars

**Figure 5:**
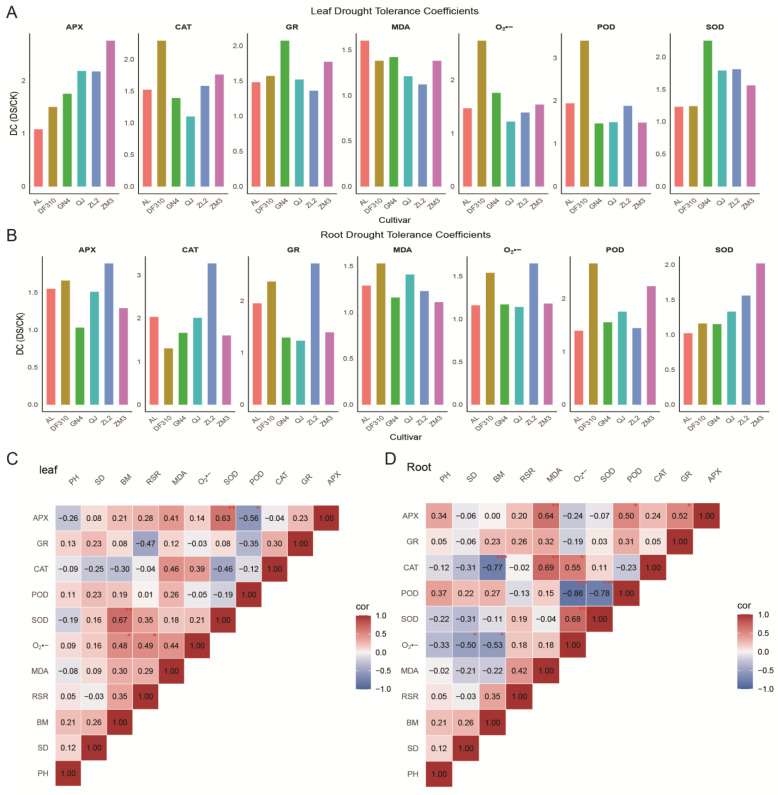
Drought tolerance coefficients and correlation of physiological indexes in alfalfa cultivars under drought stress. (**A**) Drought tolerance coefficients of leaf. (**B**) Drought tolerance coefficients of root. (**C**) Drought tolerance correlation of leaf. (**D**) Drought tolerance correlation of root. PH, plant height; SD, crown width; BM, biomass; RSR, root–shoot ratio; MDA, malondialdehyde; SS, soluble sugar; SOD, superoxide dismutase; POD, peroxidase; CAT, catalase; GR, glutathione reductase; APX, ascorbate peroxidase. O_2_•−, superoxide anion. * indicates a significant association at the 0.05 level, *, *p* < 0.05; **, *p* < 0.01; ***, *p* < 0.001.

The drought tolerance coefficients for each indicator were calculated. The activities of MDA, O_2_•^−^, SOD, POD, CAT, GR, and APX all increased in DS compared with those in CK (Figure 5A,B). To further analyze the correlations between biochemical indicators and growth traits, as shown in Figure 5C, SOD was significantly positively correlated with biomass (*p* < 0.05) in alfalfa leaves. O_2_^−^ was also significantly positively correlated with biomass and RSR. Additionally, APX showed a significant positive correlation with SOD but a significant negative correlation with POD. In roots, O_2_•^−^ was significantly negatively correlated with SD and biomass; CAT was significantly negatively correlated with biomass, but significantly positively correlated with MDA and O_2_•^−^; POD was significantly negatively correlated with O_2_•^−^ and SOD; APX was significantly positively correlated with MDA, POD, and GR (Figure 5D).

**Figure 6:**
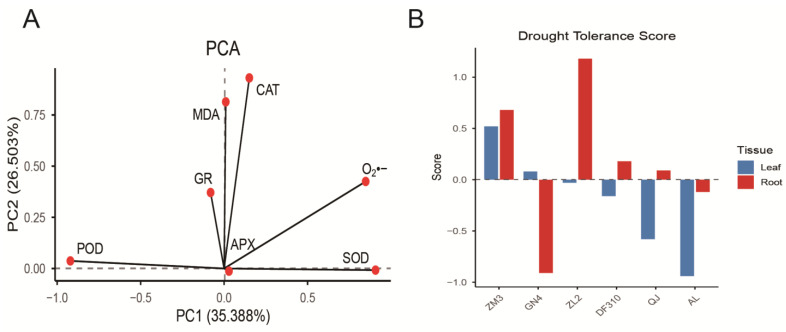
Contribution of physiological indexes and comprehensive drought tolerance assessment in alfalfa cultivars under drought stress. (**A**) Contribution of physiological indexes. (**B**) Comprehensive drought tolerance assessment. MDA, malondialdehyde; SS, soluble sugar; SOD, superoxide dismutase; POD, peroxidase; CAT, catalase; GR, glutathione reductase; APX, ascorbate peroxidase. O_2_•^−^, superoxide anion.

Additionally, principal component analysis (PCA) was performed on combined leaf and root data using the seven biochemical indicators (MDA, O_2_•^−^, SOD, POD, CAT, GR, APX) (Figure 6A). The first two principal components contribution rates were 35.388% and 26.503%, respectively. The cumulative contribution rate reached 61.891%, meeting the screening criteria for PCA. Comprehensive evaluation revealed that under the same treatment conditions, the drought tolerance ranking of the six alfalfa cultivar leaves, from highest to lowest, was: ZM3 > GN4 > ZL2 > QJ > DF310 > AL. In roots, the ranking was: ZL2 > ZM3 > DF310 > GN4 > QJ > AL (Figure 6B). Thus, ZM3 and ZL2 exhibited the highest drought tolerance in both leaves and roots.

### 3.5. Transcriptome Sequencing and Differentially Expressed Gene (DEG) Analysis

**Figure 7:**
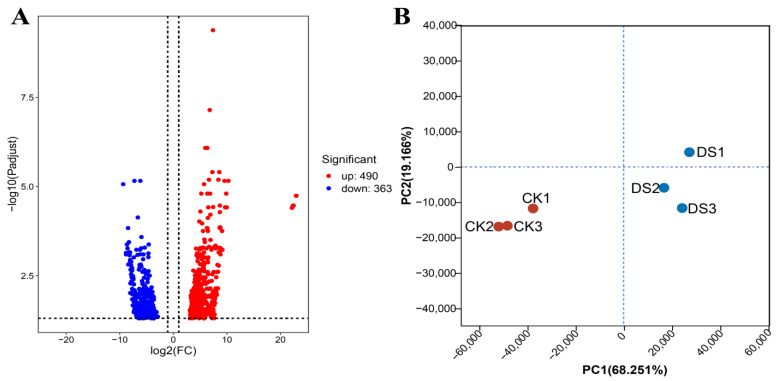
Gene expression patterns in alfalfa leaves under drought stress; (**A**) Volcano plot of DEGs. The abscissa represents the log_2_ fold change between the DS and CK groups, while the ordinate represents the negative −log_10_ adjusted *p*-value (FDR). Red and blue dots indicate significantly upregulated and downregulated genes (FDR < 0.05). (**B**) PCA. PC1 and PC2 represent the first and second principal components, with their contributions to sample variance indicated as percentages in parentheses. Red dots represent the CK sample, and blue dots represent the DS samples.

To investigate the gene expression patterns of alfalfa in response to drought stress, we performed RNA sequencing analysis. High-quality reads were obtained, and after quality control filtering, the average Q20 and Q30 values were 98.17% and 94.99%, respectively. The mean GC content as 45.02%, indicating that the sequencing data were useful for further analysis (Supplementary Table S1). First, we comprehensively examined the mRNA expression levels of alfalfa in CK and DS. A total of 853 DEGs (490 upregulated and 363 downregulated) with FDR < 0.05 were identified in the DS vs. CK comparison (Figure 7A, Supplementary Table S2). PCA based on the extracted DEGs indicated that the DS and CK groups were each divided into two subgroups (Figure 7B), demonstrating high reliability and reproducibility. These results confirmed that the sequencing data could be used for subsequent analysis, including GO and KEGG analysis and compared the expression level of the DEGs.

**Figure 8:**
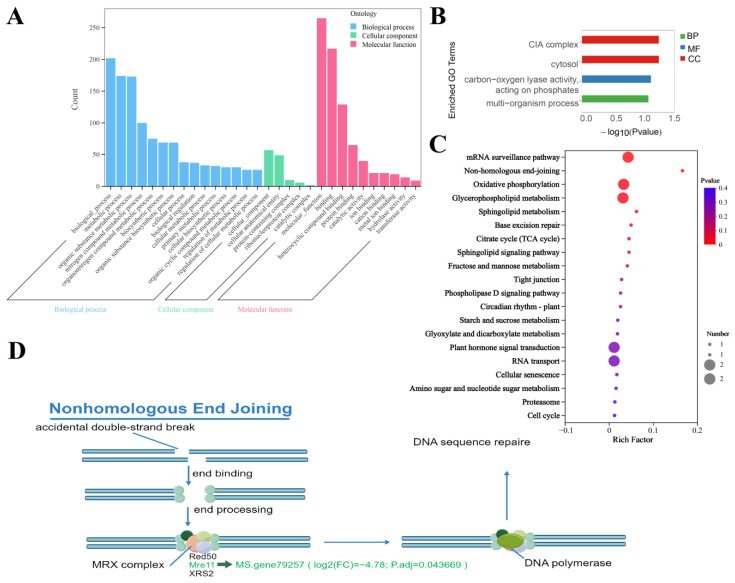
GO and KEGG enrichment classification histogram. (**A**,**B**) GO enrichment bar chart. The vertical axis represents the number of DEGs (count), while the horizontal axis lists the GO terms; and. (**C**) KEGG enrichment bubble chart. The ordinate represents the pathway, while the abscissa represents the enrichment factor (ratio of the number of DEGs in a pathway to the total number of genes in that pathway). The bubble size indicates the number of DEGs, and the color gradient represents the adjusted *p*-value (the redder the color, the smaller the *p*-value). (**D**) Schematic diagram of the Non-homologous end-joining pathway.

GO and KEGG enrichment analyses were performed to explore the functional implications of the DEGs. A total of 153 GO terms were enriched, including 98 (64.05%) under the biological process (BP) category, 43 (28.10%) under molecular function (MF), and 12 (7.84%) under cell component. Most DEGs were associated with BP and MF categories. Within the BP group, DEGs were mainly enriched in “biological process,” “metabolic process,” and “organic substance metabolic process”. Within the MF group, they were primarily enriched in “molecular function,” “binding,” and “heterocyclic compound binding” (Figure 8A). Further analysis revealed significant enrichment in four GO terms: cytosol (GO:0005829); CIA complex (GO:0097361); carbon-oxygen lyase activity, acting on phosphates (GO:0016838); multi-organism process (GO:0051704) (Figure 8B). KEGG pathway analysis identified 42 pathways in the DS vs. CK comparison. Among these, the mRNA surveillance, Non-homologous end-joining, oxidative phosphorylation, and glycerophospholipid metabolism pathways were significantly enriched (Figure 8C). Among them, the rich factor of the Non-homologous end-joining pathway was the highest among all pathways, and this pathway is closely related to the repair of DNA damage induced by stress (Figure 8D).

**Figure 9:**
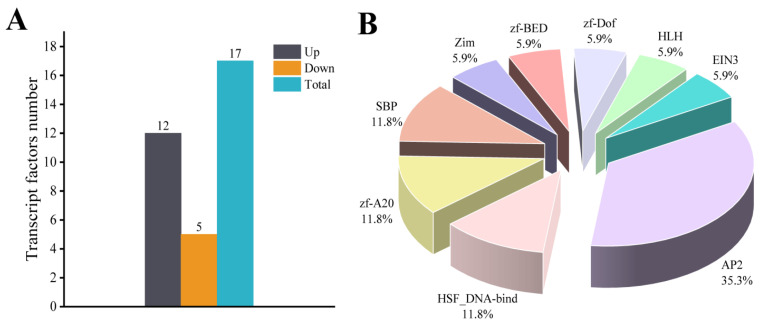
Distribution of transcription factor genes responsive to drought stresses. (**A**) Number of DEGs encoding TFs. (**B**) Classification of TFs under drought stress.

Among the 853 DEGs, 17 were annotated as transcription factors (TFs), belonging to nine families (12 up-regulated and 5 down-regulated) (Figure 9A), including AP2 (e.g., ERF3/ERF12/ERF107/EREBP), HSF_DNA bind (e.g., HSPA-6B), zf-A20, SBP (e.g., SPL8), Zim (e.g., JAZ), zf-BED, zf-Dof, HLH (e.g., MYC2), and EIN3 transcription factor families (Figure 9B), The major transcription factor families three were AP2 (35.3%), HSF_DNA bind (11.8%), zf-A20 (11.8%), SBP (11.8%). These results provide a preliminary indication of potential transcriptional regulators involved in drought response in ZM3, warranting further investigation into their functional roles.

### 3.6. Validation of Candidate Genes

**Figure 10:**
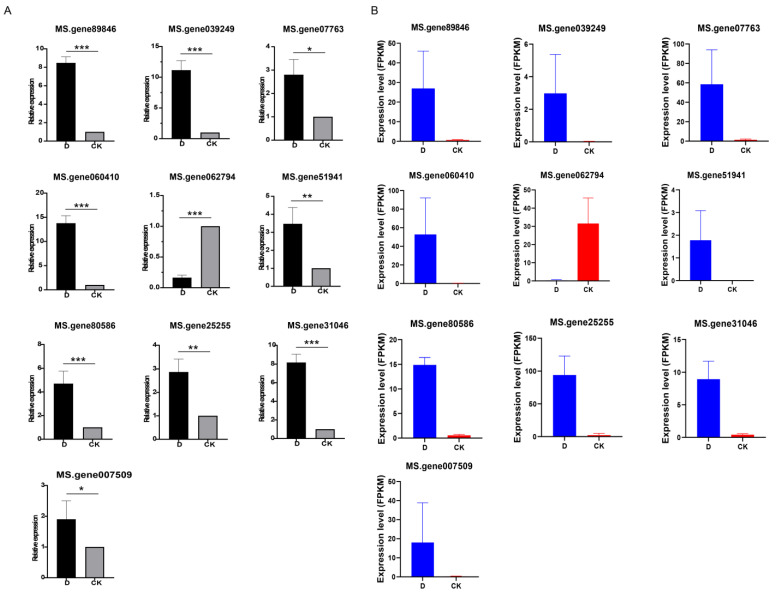
Expression pattern of the selected 10 genes. (**A**) Gene expression profiles obtained via qRT–PCR. Star indicate significant differences between the CK and treatments as determined by Student’s *t*-test. Error bars indicate the SD of three biological replicates. * *p* < 0.05; ** *p* < 0.01; *** *p* < 0.001. (**B**) Gene expression profile detected by RNA-seq.

qRT–PCR was employed to validate the reliability of our RNA-seq data. The results demonstrated that the expression patterns of ten selected DEGs were consistent with those obtained from the RNA-seq datasets (Figure 10). Among these genes, nine were significantly up-regulated in tissues following drought stress. For instance, MS.gene89846 (U-box domain-containing protein 17) and MS.gene039249 (disease resistance protein At4g27220) may serve as important candidates for further studies on drought tolerance in alfalfa.

## 4. Discussion

Drought stress has been reported to cause stomatal closure, reduced biomass yield, and delayed crop growth and development [30]. Schubert et al. found that drought stress reduced alfalfa plant height, branch number, and both above- and below-ground biomass [31]. In this study, drought significantly reduced alfalfa stem diameter and biomass, which is consistent with those findings and further confirms the negative impact of drought on alfalfa growth. However, the reduction in plant height was relatively small, aligning with the results of Zhang et al. on drought-stressed *Jerusalem artichoke* seedlings, although notable differences were observed among alfalfa cultivars [32].

Under certain drought conditions, plants typically exhibit self-regulation under certain drought conditions to adapt to environmental stress. In this study, the RSR of alfalfa was significantly higher under drought stress than that in the control group. This observation aligns with previous findings that the RSR of forage grasses such as ryegrass (*Lolium perenne* L.), tall fescue (*Festuca arundinacea* Schreb.), and chicory (*Cichorium intybus* L.) increases under drought conditions [33]. This response may be attributed to the differential effects of drought stress on plant roots and leaves. Under drought stress, rapid osmotic regulation and enhanced water-use efficiency in the root system promote root growth over leaf growth. Consequently, alfalfa allocates more biomass to the root system, increasing its RSR to obtain more limited nutrients, which is a key adaptive mechanism contributing to drought tolerance [34]. However, the adverse effects of drought stress depend on its severity and duration, as well as on the growth stage of the crop. Therefore, future studies should continue monitoring changes in aboveground growth, water transport, and water-use efficiency in alfalfa under varying drought intensities and durations to further elucidate the impact of drought on alfalfa growth.

MDA is a physiological indicator reflecting the extent of membrane lipid peroxidation under abiotic stress and serves as an important marker of plant stress. Its concentration reflects the degree of peroxidation damage to the plasma membrane [35]. As an end product of lipid peroxidation caused by ROS accumulation, MDA can bind to membrane proteins and enzymes, disrupting protein cross-linking and thereby impairing membrane structure and function [36]. Higher MDA levels indicate more severe plant damage, with sensitive cultivars exhibiting severe disruption of the membrane system and increased cell membrane permeability [37]. In this study, both MDA and O_2_^−^ levels significantly increased in all six alfalfa seedling cultivars under drought stress (*p* < 0.05 or *p* < 0.01). This indicates that drought stress induces lipid peroxidation and oxidative damage in alfalfa seedlings, causing considerable damage to the cell membrane. Cell membrane permeability may therefore serve as an effective indicator for assessing drought tolerance in alfalfa cultivars. Similarly, MDA content in the root systems of potato seedlings gradually increased under prolonged drought, indicating metabolic imbalance and enhanced lipid peroxidation in root cell membranes [38]. Thus, MDA is a reliable measure of lipid peroxidation intensity and the extent of plasma membrane damage, with its content reflecting the severity of stress-induced damage in plants. The higher the MDA content, the greater the damage [39]. In this study, the MDA and O_2_^−^ contents of all alfalfa cultivars increased under drought stress, consistent with the findings of Zhang et al. in purple alfalfa and Wang et al. in *Agropyron mongolicum* [40,41].

Under normal conditions, the production and removal of ROS within cells are in dynamic equilibrium. Drought stress, however, leads to excessive ROS accumulation in plants, disrupting cell membrane stability. Plants mitigate this oxidative damage through their antioxidant enzyme system, which removes excess ROS [42]. Key enzymes in this system include SOD, POD, CAT, GR, and APX [43]. SOD catalyzes the dismutation of superoxide radicals within cells and plays a central role in plant ROS metabolism. POD and CAT reflect physiological and biochemical processes, decomposing harmful H_2_O_2_ within cells into water and oxygen, thereby protecting cells against ROS damage and serving as markers of plant stress resistance [44,45]. Different plants utilize distinct sets of enzymes to protect themselves and mitigate the impact of external environmental factors.

Wang et al. found that in alfalfa seedlings under drought and salt stress, the antioxidant enzyme system reduces ROS accumulation, with enzyme activity increasing under drought conditions [46]. Similarly, in this study, we found that SOD, POD, CAT, GR, and APX activities in the leaves and roots of alfalfa seedlings increased under drought stress. This suggests that alfalfa mitigates drought-induced damage by increasing the activities of these enzymes in the leaves and roots. Collectively, these enzymes eliminate superoxide radicals converted into H_2_O_2_, thereby reducing membrane lipid peroxidation and protecting internal tissues while maintaining normal cellular membrane metabolism. These findings are consistent with previous studies on quinoa and naked oat [47,48]. Thus, the antioxidant enzyme system in seedling leaves and roots is sensitive to drought conditions, and the trends among different enzymes are generally consistent. This suggests that the enzymes in the plant antioxidant enzyme system function as an integrated network, requiring mutual collaboration to exert protective effects. This aligns with the findings of Liu et al. in *Hippophae rhamnoides* [49].

Understanding drought tolerance in alfalfa is crucial for improving its resilience and developing drought-tolerant cultivars. Drought tolerance is a complex, multifaceted trait influenced by various factors. Individual indicators respond differently to drought stress, and relying on a single indicator cannot provide an accurate assessment. Therefore, a multi-indicator comprehensive evaluation is necessary [50]. Multivariate analysis methods such as PCA and membership function analysis are reliable and effective evaluation methods widely used in screening stress-tolerant alfalfa cultivars [51]. In this study, PCA was used to condense the drought tolerance coefficient values of seven indicators into three comprehensive indicators. Subsequently, membership function analysis was employed to calculate the comprehensive drought tolerance scores for each cultivar, and the cultivars were ranked based on these scores. The drought tolerance of alfalfa seedlings’ leaves was ranked as follows: Zhongmu No.3 > Gannong No. 4 > Zhonglan No.2 > Qiji > Algonquin > DF310. The drought tolerance of roots was ranked as follows: Zhonglan No.2 > Zhongmu No.3 > DF310 > Qiji > Algonquin > Gannong No.4. Selecting different tissue indicators may yield different results. Therefore, further research on additional varieties under drought stress should investigate alfalfa’s drought tolerance in terms of cellular structure, osmotic regulatory substances, endogenous hormones, and root system architecture. This would enable a more comprehensive analysis of drought tolerance physiology in alfalfa varieties, from above-ground to below-ground and from tissue structure to physiological and biochemical aspects.

Transcriptomics has recently emerged as a key approach in identifying stress-resistant genes and elucidating plant stress response mechanisms. Therefore, this study employed transcriptomic sequencing to systematically analyze the gene expression profile of the drought-resistant alfalfa cultivar ZM3 under drought stress, identifying a large number of DEGs. GO functional enrichment analysis of these DEGs revealed significant enrichment in biological processes and cellular components such as cytosol (GO:0005829); CIA complex (GO:0097361); carbon-oxygen lyase activity, acting on phosphates (GO:0016838); and multi-organism process (GO:0051704). This suggests that the cultivar may counteract drought stress by enhancing ROS scavenging capacity and signal transduction efficiency. KEGG pathway analysis further revealed that DEGs were predominantly enriched in pathways including the “mRNA surveillance pathway,” “Non-homologous end-joining”, “oxidative phosphorylation,” and “glycerophospholipid metabolism”. These KEGG pathways are all closely associated with plant drought resistance. They assist plants in coping with drought stress through regulation of mRNA stability, energy supply, and maintenance of cell membrane integrity. The accumulation of osmotic regulatory substances and cell wall modifications may also constitute key mechanisms underpinning its drought resistance.

The non-homologous end-joining pathway, which is critical for DNA double-strand break repair, was significantly enriched in the drought-tolerant cultivar ZM3 (Figure 8C). As a core component of this pathway, MRE11 has been reported to participate in stress responses in plants. For example, in *Medicago truncatula*, MtMRE11 was upregulated in transgenic plants with enhanced osmotic stress tolerance [52], and another study directly demonstrated that MtMRE11 is upregulated in response to γ-ray-mediated genotoxic stress [53]. However, in the present study, MRE11 expression was downregulated in ZM3 after drought treatment. This discrepancy suggests that the transcriptional regulation of MRE11 under drought is complex and context-dependent. The downregulation may reflect a negative feedback mechanism following DNA repair, or post-transcriptional regulation that maintains protein function despite reduced transcript abundance. Further studies are needed to clarify the role of MRE11 in alfalfa drought tolerance.

Additionally, multiple significantly upregulated transcription factor families (e.g., ERF, MYC, HSP, and JAZ) were identified. These transcription factors play central roles in regulating stress response gene expression and likely establish an efficient drought-tolerance regulatory network by synergistically activating downstream stress-resistant functional genes. In summary, this stress-resistant variety exhibits significantly enhanced adaptability to drought stress through a complex regulatory mechanism involving the coordinated participation of multiple genes and pathways. This provides a theoretical basis and candidate gene resources for further improving crop stress tolerance using molecular breeding approaches.

We acknowledge that certain widely used physiological indicators, such as relative water content, chlorophyll content, photosynthetic rate, and leaf area, were not measured in this study. These parameters would provide additional insights into leaf-level water status and photosynthetic efficiency under drought. Additionally, our transcriptomic analysis was confined to leaf tissues of the drought-tolerant cultivar ZM3; root transcriptomes were not examined. Therefore, the molecular basis of the coordinated leaf–root antioxidant response observed physiologically remains to be elucidated in future studies.

## 5. Conclusions

This study investigated the effects of drought stress on the growth and leaf/root physiology of six alfalfa cultivars with varying levels of drought tolerance and comprehensively evaluated their drought resistance. Drought stress affected alfalfa growth, resulting in a decrease in biomass. Under drought conditions, all six alfalfa cultivars exhibited increased activity of antioxidant enzymes such as SOD, POD, CAT, GR, and APX in both leaves and roots. These enzymes worked synergistically to improve plant survival under stress. However, the key enzymes involved varied among different cultivars, indicating that the underlying mechanisms require further investigation. A comprehensive evaluation of the six alfalfa cultivars revealed the following rankings for leaf drought tolerance: for leaves, ZM3 > GN4 > ZL2 > QJ > DF310 > AL; for roots, ZL2 > ZM3 > DF310 > GN4 > QJ > AL. These findings provide a theoretical and experimental basis for the selection and breeding of drought-tolerant alfalfa cultivars, although limitations such as missing physiological parameters and root transcriptome data remain to be addressed.

## Data Availability

RNA-seq raw data are available at the National Center for Biotechnology Information (NCBI) Sequence Read Archive (SRA) database (accession number PRJNA1051509).

## Supplementary Materials

The following supporting information can be downloaded at: https://www.mdpi.com/article/10.3390/plants15111737/s1, Supplementary Table S1. Statistics of RNA-seq information**;** Supplementary Table S2. The DEGs list between CK and DS.
